# Prediction of Detailed Enzyme Functions and Identification of Specificity Determining Residues by Random Forests

**DOI:** 10.1371/journal.pone.0084623

**Published:** 2014-01-08

**Authors:** Chioko Nagao, Nozomi Nagano, Kenji Mizuguchi

**Affiliations:** 1 National Institute of Biomedical Innovation, Ibaraki, Osaka, Japan; 2 Computational Biology Research Center, AIST, Koto-ku, Tokyo, Japan; University of Florida, United States of America

## Abstract

Determining enzyme functions is essential for a thorough understanding of cellular processes. Although many prediction methods have been developed, it remains a significant challenge to predict enzyme functions at the fourth-digit level of the Enzyme Commission numbers. Functional specificity of enzymes often changes drastically by mutations of a small number of residues and therefore, information about these critical residues can potentially help discriminate detailed functions. However, because these residues must be identified by mutagenesis experiments, the available information is limited, and the lack of experimentally verified specificity determining residues (SDRs) has hindered the development of detailed function prediction methods and computational identification of SDRs. Here we present a novel method for predicting enzyme functions by random forests, EFPrf, along with a set of putative SDRs, the random forests derived SDRs (rf-SDRs). EFPrf consists of a set of binary predictors for enzymes in each CATH superfamily and the rf-SDRs are the residue positions corresponding to the most highly contributing attributes obtained from each predictor. EFPrf showed a precision of 0.98 and a recall of 0.89 in a cross-validated benchmark assessment. The rf-SDRs included many residues, whose importance for specificity had been validated experimentally. The analysis of the rf-SDRs revealed both a general tendency that functionally diverged superfamilies tend to include more active site residues in their rf-SDRs than in less diverged superfamilies, and superfamily-specific conservation patterns of each functional residue. EFPrf and the rf-SDRs will be an effective tool for annotating enzyme functions and for understanding how enzyme functions have diverged within each superfamily.

## Introduction

Almost all chemical reactions in living organisms are catalyzed by enzymes [1]. For a thorough understanding of cellular processes, it is essential to determine enzyme functions, i.e., what types of reactions are catalyzed, and what chemical compounds are utilized as substrates or cofactors. Prediction of enzyme function is a longstanding problem and many methods have been developed. The targeted functional details range from the broadest classification level such as enzyme/non-enzyme discrimination to a highly specific scheme such as the four-digit Enzyme Commission (EC) numbers [2]. Also, different types of features have been used, such as sequence/structural similarities, physico-chemical properties of amino acids, specific sequence/structural motifs, and their combinations [3]–[12]. Furthermore, many methods have been proposed recently for large-scale prediction of protein functions defined by Gene Ontology (GO) terms [13]. However, the most widely used method for functional annotation remains the simplest one: the transfer of functions based on sequence similarity calculated by BLAST/PSI-BLAST [14], [15], despite its known limitations [16]–[19]. Moreover, predicting a precise enzyme function is still a significant challenge, as only a few methods currently available can predict the full four-digit EC numbers. The knowledge of such detailed functions can help determine true substrates for disease-related enzymes and design specific inhibitors for drug targets.

Enzymes in a protein family are considered to be evolutionary related. In many cases, these enzymes have similar but different functions. Divergence of sequences and functions are different in each family. Some enzymes, which share the sequence identity of over 90%, have different functions and differ in the first-digit of their EC numbers [16]–[19]. On the other hand, some enzymes, the sequence identity of which is below 30%, share all four digits of the EC numbers. This nonlinear correlation between function and sequence similarity makes the identification of detailed functions of enzymes such a difficult task.

One solution to overcome this problem is to use the information about functionally critical residues. The construction and use of sequence motifs can be considered an example of this approach [20], [21]. Residues critical for functions, mutations of which bring drastic changes in the catalytic efficacy or substrate specificity, are sometimes called specificity determining residues (SDRs) or function determining residues (FDRs). Proper information about SDRs is expected to improve the ability to distinguish enzyme functions [22]–[24]. However, such information is limited, because SDRs are determined by mutagenesis experiments. Therefore, most prediction methods use other properties serving as a proxy for SDRs [4], [6], [23]–[26]: catalytic residues, ligand binding sites or residues conserved in a functional subfamily. The lack of information about SDRs has hindered the development of computational methods for identifying SDRs [27]–[30] as well as predicting detailed functions.

Some machine learning methods can construct classifiers from a large number of attributes and calculate contributions from each attribute. Random forests [31] are one of the most accurate machine learning algorithms used for many applications, including the analysis of microarray data [32], [33] and prediction of protein-protein interactions [34], [35]. For enzyme function prediction, random forests have been applied for assigning the first or second digit of the EC numbers [7], [8], [36], [37]. These methods used several hundreds of physico-chemical features calculated from only the full-length sequences and thus, provided no information about the importance of each residue for discriminating different functions.

In this study, we applied random forests, for the first time, for predicting the four-digit EC numbers (rather than only the first or second digit) in each homologous superfamily and also for obtaining a putative set of SDRs at the same time by using residue position specific attributes. We focus on a problem of discriminating detailed enzyme functions within a single protein family, since methods for assigning a protein sequence to an existing family have been well established. Thus, we assume that a functionally unknown protein has been already classified into a known protein family by sequence similarity. Given this framework, our objectives were two-fold; first, we aimed to develop a method that can predict the full four-digit EC number for a given protein. Second, we aimed to define putative SDRs as the most highly contributing positions used in our prediction model. Characterizing these “computational defined SDRs” in a systematic manner should mitigate the lack of experimentally defined SDRs.

Our analysis is based on the CATH domain classification [38]; we created a dataset from the UniProtKB/Swiss-Prot database [39] by selecting the enzymes, which had complete four-digit EC numbers and for which CATH homologous superfamilies were assigned by Gene3D [40]. For each enzyme in each superfamily, binary predictors were constructed by random forests with full-length sequence similarities and the residue similarities for active sites, ligand binding sites and conserved sites as input attributes. From the most highly contributing attributes, we obtained a set of putative SDRs and termed them random forests derived SDRs (rf-SDRs). The predictors (EFPrf) showed a performance comparable to that of a related method currently available and the rf-SDRs included many residues, for which functional importance had been verified by experimental studies. This study revealed a general tendency that functionally diverged superfamilies tend to include more active site residues (ASRs) in their rf-SDRs than in less diverged superfamilies. From the analysis of selected superfamilies, we also made superfamily-specific observations that conserved residues across enzymes, even if functionally important, tend not to be selected as rf-SDRs.

## Results and Discussion

### Overview of the enzyme function prediction

**Figure 1:**
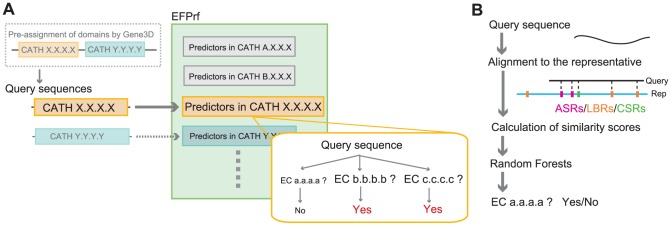
Outline of the EFPrf system (A) and the predictor for each enzyme constructed by Random Forests (B). A query to the system is a domain sequence pre-assigned to a CATH homologous superfamily by Gene3D. For each CATH superfamily, binary predictors, each for a known enzyme, process the query and return their results (A). In each predictor, the query is aligned to a representative sequence by the FUGUE software. Based on the alignment, similarity scores for the full-length sequence and at the functional sites are calculated for the input to the predictor (B).

Figure 1A describes an overview of the enzyme function prediction method by random forests (EFPrf). A query to the system is a domain sequence pre-assigned to a CATH homologous superfamily (indicated as CATH X.X.X.X in the figure) by Gene3D. We chose a CATH homologous superfamily as a unit of protein family because a structure-based classification scheme can capture more distant proteins than a sequence-based one. In CATH X.X.X.X superfamily, binary predictors for each enzyme have been developed (Figure 1B). In each predictor, the query is aligned to the representative sequence by the FUGUE software [41] with the structure environment-specific substitution tables (ESSTs). Based on the alignment, the similarity scores for the full-length sequence and at the functional sites are calculated for the input to the predictor.

### Dataset construction

**Figure 2:**
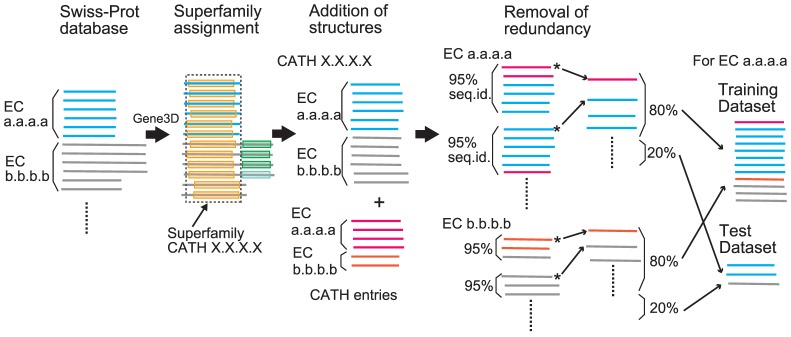
Outline of dataset construction. From the UniProtKB/Swiss-Prot database, the enzyme sequences, for which complete EC numbers are assigned, were obtained and their CATH domain regions from the Gene3D database were selected. After adding CATH entries and removal of redundancies, the enzymes having less than ten sequences were removed. The representative structures for each enzyme were selected from the CATH S-level representatives. In the remaining sequences, a predictor was constructed for an enzyme, which has sufficient numbers of positive and negative sequences (see Materials and Methods for more details). Randomly selected 80% of the sequences were used for training. The remaining 20% of the sequences were used as a test dataset.

We selected the enzyme sequences from the UniProtKB/Swiss-Prot database, for which complete EC numbers are assigned, and obtained their CATH domain regions from the Gene3D database. After removing redundancies, predictors have been constructed for the enzymes that had ten or more sequences and had at least one other enzyme in the superfamily (with a total of ten or more sequences) as negative data (Figure 2; see Materials and Methods for more details). Thus, we have built predictors for 1121 enzymes distributed over 306 CATH superfamilies. The representative structures for each enzyme were selected from the CATH S-level representatives with the longest sequence length and the highest resolution. In each superfamily, 3.7 enzymes were selected for constructing predictors on average. In 89 superfamilies, a single predictor was constructed. Fifteen superfamilies contained more than ten enzyme predictors and the largest superfamily was the NAD(P)-binding Rossmann-like domain superfamily (CATH 3.40.50.720) with 65 predictors (Table S1 and Figure S1). All the superfamilies, for which at least one predictor was created, were included in the analysis below.

### Additional information to BLAST score improved the precision of the prediction

To investigate whether the use of the information about functional residues improves prediction performance or not, we built two types of predictors. First, we created simple decision trees by C4.5 with the BLAST bit score for the top hit in each enzyme as an attribute (“the simple model”). Because BLAST scores are the most widely used measure for function transfer, the simple model served as our baseline for predicting enzyme functions. Next, we constructed a second set of predictors by random forests (EFPrf) with more attributes. Three scoring matrices, BLOSUM62 [42], position specific scoring matrices (PSSM) [43] and ESST-based structural profiles, were used to calculate the scores at the active site residues (ASRs), ligand binding residues (LBRs) and conserved residues (CSRs), in addition to the full-length scores. The resulting 12 ( = 3×4) attributes and the BLAST score were used as input to the system.

**Figure 3:**
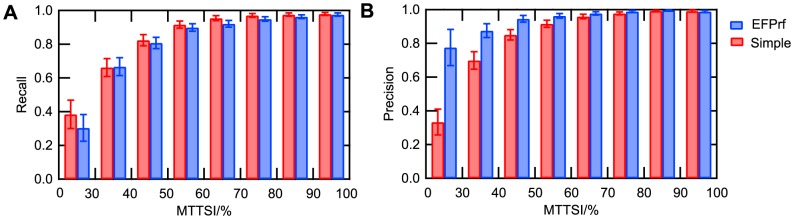
Prediction performance of EFPrf. The recall (A) and precision (B) at each level of the maximal test to training sequence identity (MTTSI) are plotted for the simple model (red) and the EFPrf (blue). Error bars represent 95% confidence intervals in each MTTSI range.

In a cross-validated benchmark assessment (see Materials and Methods), we followed a previous study [4] and calculated the maximal test to training sequence identity (MTTSI) for each query, and evaluated the prediction performance for eight different MTTSI ranges separately. Figure 3 and Table S2 show recall and precision averaged in each of the eight MTTSI ranges. (The average was taken by using only the enzymes, for which precision or recall was defined in the given MTTSI range.) In Figure 3A, recall in all ranges shows no significant differences between the simple model and EFPrf. On the other hand, precision improved significantly by EFPrf, especially in the lowest MTTSI range, where distinguishing functions by sequence similarity alone is known to be difficult (Figure 3B). This result indicates that the additional information about functionally important residues is useful for discriminating detailed functions. Table 1 shows the prediction performance averaged over the 1121 enzyme predictors (see Table S3 for the individual values). Although a general trade-off between recall and precision was observed, the statistically significant increase in the F-measure achieved by EFPrf over the simple model also suggested the usefulness of the additional attributes of ASRs/LBRs/CSRs.

**Table 1:** Prediction performance.

Model	Precision	Recall	F-measure
Simple	0.94	0.91	0.92
EFPrf	0.98 (<2.2e-16)	0.89 (1.3e-5)	0.93 (0.009)

The values in the parentheses represent the p-values calculated against the simple model by paired t-test.

Because of differences in the training and test datasets, a direct comparison of performance with other methods is difficult but the prediction performance of EFPrf (recall = 0.30, precision = 0.78 in MTTSI <30%) is comparable to or better than that of EFICAz^2^ [4], [5] (recall = 0.23, precision = 0.74 in MTTSI <30%), which combines FDRs recognition, sequence similarity and support vector machine (SVM) models. Moreover, EFICAz^2^ and EFPrf achieved an average precision of above 0.9 for MTTSI ≥40%, which is considered to be a “non trivial achievement” [4], [17].

### General properties of the random forest derived SDRs

In constructing the EFPrf, importance scores for each attribute were also calculated. We selected the top 3×√*n* attributes as “highly contributing attributes”, where *n* is the number of input attributes for each enzyme, and defined the residue positions in the highly contributing attributes (except for the full-length sequence similarity score) as the “random forests derived SDRs” (rf-SDRs) (Table S4). (In all enzymes, the full-length sequence similarity score was included in the highly contributing attributes, consistent with the result that the simple model was a modestly successful predictor.) On average, 8.4 residue positions were selected as the rf-SDRs for each enzyme. Among the position specific attributes calculated with different scoring matrices, the most frequently selected were those with PSSMs, suggesting that PSSMs may represent the amino acid differences among enzymes having similar structures/functions more clearly than the other scoring matrices (Table S5).

**Figure 4:**
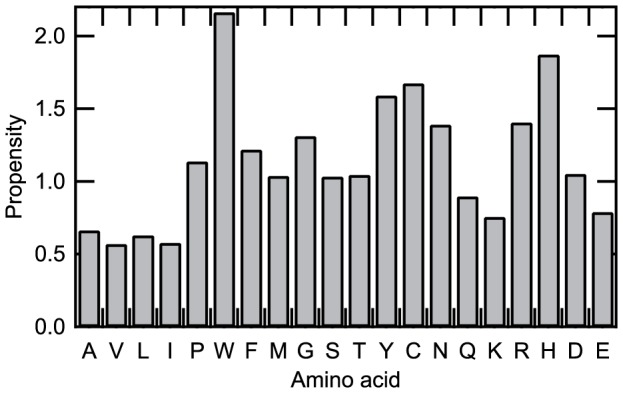
Amino acid propensities for the rf-SDRs. The propensity of amino acid *i* was calculated as the fraction of amino acid *i* in the rf-SDRs divided by the fraction of amino acid *i* in all representative enzyme domains.

Figure 4 shows the amino acid propensity for the rf-SDRs. The propensity of amino acid *i* was obtained as the fraction of amino acid *i* in the rf-SDRs divided by the fraction of amino acid *i* in all representative enzyme domains. In general, polar or charged residues were overrepresented in the rf-SDRs and non-polar residues were underrepresented. In polar, aromatic and charged residues, Trp, Tyr, Cys, Asn, Arg and His had a particularly high propensity value and in non-polar hydrophobic residues, Ala, Val, Leu and Ile had a low propensity value. In charged residues, Lys and Glu were underrepresented. This biased distribution of charged residues suggests that the delocalized charge in the guanidino group of Arg may be better utilized for SDRs than the charge in Lys, as observed in protein-protein interactions [44], and that the short side chain of Asp, with a smaller degree of freedom than that for Glu, is more suitable to form specific interactions. Some of the propensity values are different from those observed in the Catalytic Site Atlas (CSA) [45]; Asn favored for non-catalytic sites in the CSA [46], was overrepresented in the rf-SDRs and Lys and Glu, favored for catalytic sites in the CSA, were underrepresented. These differences are likely due to different definitions of functional residues, because the rf-SDRs were selected from not only catalytic sites but also ligand binding and conserved sites.

To analyze the relationships between functional diversity and the residues important for distinguishing functions, we classified superfamilies based on the functional entropy, defined by using the number of distinct EC numbers up to the third- and forth-digit levels (see details in Materials and Methods; Table S6). In the third-digit level classification, the three classes defined, the low-, medium- and high-degrees of functional diversity, approximately corresponded to having one, two to four, and more than four distinct EC numbers at the third-digit level within each superfamily. In the fourth-digit level classification, the low-, medium- and high-degrees of diversity corresponded to having one to five, six to ten and more than ten distinct EC numbers at the fourth-digit level within each superfamily. The prediction performance for the most diverged class was shown to be lower than that for the other classes in both the third- and fourth-digit based classification schemes (Tables S7 and S8).

**Figure 5:**
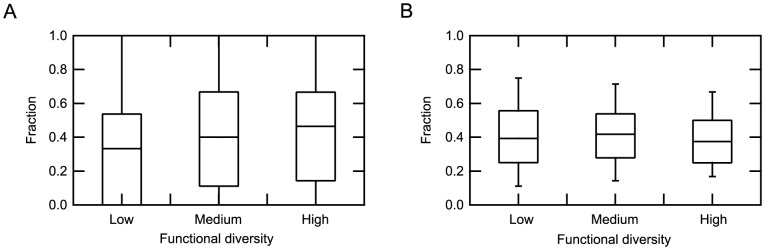
Distributions of fractions of the rf-SDRs in active site residues (ASRs, A) and ligand binding residues (LBRs, B), observed in the superfamilies with low, medium and high degrees of functional diversity classified at the third-digit level of EC numbers. The top and bottom of a box indicate 75th and 25th percentiles and the horizontal line in a box represents the median value. The top and bottom whiskers represent 90th and 10th percentiles.

We then decided to examine what proportion of the ASRs or LBRs were selected as rf-SDRs in each superfamily. We excluded the CSRs from this analysis, because the ASRs and LBRs should be more directly linked to enzyme functions, whereas the identification of CSRs depended on the number of available sequences. If we consider all the superfamilies, the rf-SDRs included either no ASRs, about half of them or all of them (corresponding to peaks at zero, 0.5 and one in Figure S2), while in many superfamilies, about half of the LBRs were selected to be rf-SDRs (a peak around 0.5). We next examined these quantities as a function of functional diversity. Figure 5 and Table S9 showed that the proportion of ASRs to be selected as rf-SDRs increased with functional diversity, as defined by numbers of the third-digit EC number level functions. Although this tendency was weak (with moderate statistical significance for the difference; p-value = 0.019 for the superfamilies with low and medium functional diversity, and p-value = 0.017 for those with low and high functional diversity by the Wilcoxon rank sum test), it is consistent with the notion that enzymes in a superfamily with low functional diversity often have similar active sites and similar catalytic mechanisms and thus, ASRs generally do not distinguish different functions. On the other hand, the proportion of LBRs to be selected as rf-SDRs decreased slightly from medium to high functional diversity but almost unchanged between low and high functional diversity, suggesting that LBRs can discriminate functions in superfamilies with all ranges of functional diversity. The same tendency was observed with functional diversity defined by numbers of the fourth-digit EC number level functions (Figure S3 and Table S10). The similar tendencies between the two classification schemes, observed in prediction performance and the proportions of ASRs and LBRs, may be accounted for by the observation that superfamilies with high functional diversity at the third-digit level generally have many distinct fourth digits in each third-digit EC number function.

### Examples of superfamilies and enzymes

In this section, we describe a detailed investigation of the properties of the rf-SDRs in selected enzymes from superfamilies with different degrees of functional diversity. To remove potential biases associated with protein folds, we first show three superfamilies from a single fold, and next we show an additional example from a different fold. Only three folds, TIM barrel (CATH 3.20.20), α-βplaits (CATH 3.30.70) and Rossmann fold (CATH 3.40.50), satisfied the condition of having superfamilies in each of all three classes of functional diversity and in each class, containing at least one enzyme, for which the ASR information was available. From these three, we selected the TIM barrel fold (CATH 3.20.20). The TIM barrel, (α/β)_8_-barrel fold, is one of the largest and oldest fold and in the enzymes belonging to this fold, all the active sites are located at the C-terminal ends of the β-strands. As typical examples of superfamilies with low and high functional diversity, we chose glycosidases (CATH 3.20.20.80) and aldolase class I (CATH 3.20.20.70), respectively. We then chose phosphoenolpyruvate-binding domains (CATH 3.20.20.60) as an example of the superfamilies with medium functional diversity, although the number of enzymes with available ASR information was limited and the proportion of ASRs to be selected as rf-SDRs was somewhat atypical. Therefore, we additionally examined the α/β-hydrolase superfamily (CATH 3.40.50.1820) as a second example of the superfamilies with medium diversity, because this superfamily highlighted deviations from the average properties of this class of superfamilies explained by the well conserved catalytic triad.

#### Glycosidase superfamily (CATH 3.20.20.80)

**Figure 6:**
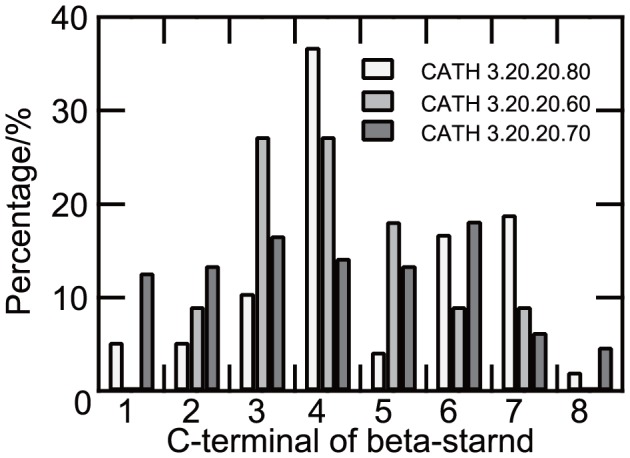
The distribution of active site residues at the end of eight β-strands of enzymes in the superfamilies adopting the TIM barrel fold. White bars represent the glycosidase superfamily (CATH 3.20.20.80), light gray bars represent the phosphoenolpyruvate-binding domain superfamily (CATH 3.20.20.60), and gray bars represent the aldolase class I superfamily (CATH 3.20.20.70). The percentages were calculated by using 18, three and 29 enzymes for glycosidases, phosphoenolpyruvate-binding domains and aldolase class I, respectively, for which active site information was available.

The glycosidase superfamily, where most enzymes belong to glycosidases (EC. 3.2.1), is a superfamily with low functional diversity. In our dataset, this superfamily contained 16 different glycosidases (EC 3.2.1) and three different hexosyltransferases (EC 2.4.1) (Table S3). The white bars in Figure 6 shows the distribution of the positions of the active site residues at eight C-terminal ends of the β-strands in this superfamily, highlighting three main catalytic residues at the β-strands 4, 7 and 6. This observation is consistent with the fact that 12 of the 16 glycosidases in this superfamily have been characterized as members of a group known as “the 4/7 group” [47]–[49]. (In the literature, this group is normally referred to as “the 4/7 superfamily” but to avoid confusion, we use the term group here.) The enzymes in the 4/7 group utilize two conserved catalytic acidic residues located at the C-terminal ends of β-strands 4 (acid/base) and 7 (nucleophile), as well as residues at the end of β-strand 6, which modulate the nucleophile. This biased distribution is reflected in the proportion of ASRs to be selected as rf-SDRs (32.7%), which was lower than the average for the group of superfamilies with low functional diversity (35.0%), (Tables S9 and S11).

**Figure 7:**
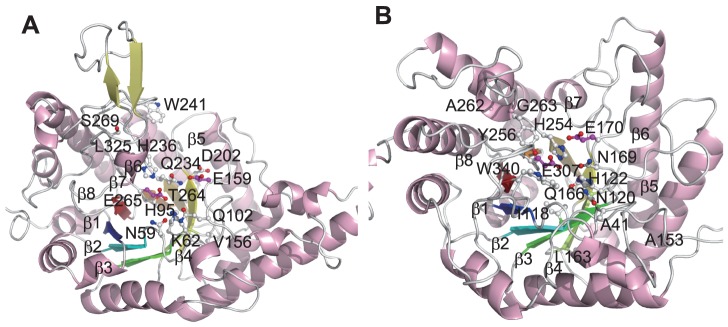
The rf-SDRs for (A) endo-1,4-xylanase (EC 3.2.1.8, CATH domain: 1r87A00) and (B) cellulase (EC 3.2.1.4, CATH domain: 1edgA00) in the glycosidase superfamily (CATH 3.20.20.80). The rf-SDRs are represented by balls and sticks, where nitrogen atoms are colored blue, oxygen atoms are red, sulfur atoms are yellow and carbon atoms are white. The carbon atoms of the active sites selected as rf-SDRs are colored magenta. Eight β-strands in a conventional barrel are colored blue, cyan, green, lemon, yellow, yelloworange, orange, and red, from the N-terminal to the C-terminal. In both enzymes, none of the two catalytic acid residues common in many enzymes in the superfamily, colored magenta, was selected.

Figure 7 shows two example enzymes of the 4/7 group, endo-1,4-β-xylanase (EC 3.2.1.8, Figure 7A) and cellulase (EC 3.2.1.4, Figure 7B). In both enzymes, none of the two 4/7 catalytic residues (Glu 159, Glu 265 in Figure 7A and Glu 170, Glu 307 in Figure 7B, respectively) was selected as the rf-SDRs. The rf-SDRs included some residues on β-strand 6, His 236 in endo-1,4-β-xylanase and His 254 and Tyr 256 in cellulase, which contact the nucleophiles and are invariant in each enzyme but different between the two enzymes [50]–[52]. The proportion of ASRs to be selected as rf-SDRs in endo-1,4-β-xylanase is lower (0.25) than that in cellulase (0.5), possibly because the former enzyme share the active site residues (other than the 4/7 catalytic residues) with a larger number of other enzymes such as glucan 1,4-α-maltohydrolase (EC 3.2.1.133) and cyclomaltodextrin glucanotransferase (EC 2.4.1.19) than the latter enzyme.

The rf-SDRs also included some LBRs, which are located in similar spatial positions but not equivalent in the sequence alignment, His 95 (endo-1,4-β-xylanase) and His 122 (cellulase) [50] shown to be essential for ligand binding by mutagenesis experiments [53]–[55], and the residues critical for determining the substrate positions, Trp 241 at the +3 subsite [56], Asn 59 and Lys 62 at the -2 subsite [57], in endo-1,4-β-xylanase.

#### Aldolase class I superfamily (CATH 3.20.20.70)

The Aldolase class I superfamily is known to be an old family including a variety of enzymes. In our dataset, predictors for 34 different enzymes were constructed in this superfamily (Table S3). These 34 enzymes included EC numbers with six different first-digits, showing the highest functional entropy in all the superfamilies. The ASR positions showed a broad distribution, indicating that the numerous functions are achieved by the active sites located at various ends of β-strands (Figure 6, dark gray bars). For instance, in 5-aminolevulinic acid dehydratase (ALADH, EC 4.2.1.24) [58], the catalytic Lys 195 and Lys 247 are positioned at the ends of β-7 and β-8, respectively and in phosphoribosylformimino-5-aminoimidazole carboxamide ribonucleotide (ProFAR) isomerase (HisA, EC 5.3.1.16) [59], the catalytic Asp 8 is positioned at the C-terminal end of β-1. Aldolase class I enzymes typically have substrates or cofactors with a phosphate-group, such as flavin mononucleotide (FMN), but enzymes in this superfamily also act on a variety of other substrates. The proportion of ASRs to be selected as rf-SDRs (51.9%) was higher than the average for the group of superfamilies with high functional diversity (43.7%) (Tables S9 and S11). This observation suggests that the ASRs located differently among the enzymes can be used effectively for discriminating different functions in this superfamily.

**Figure 8:**
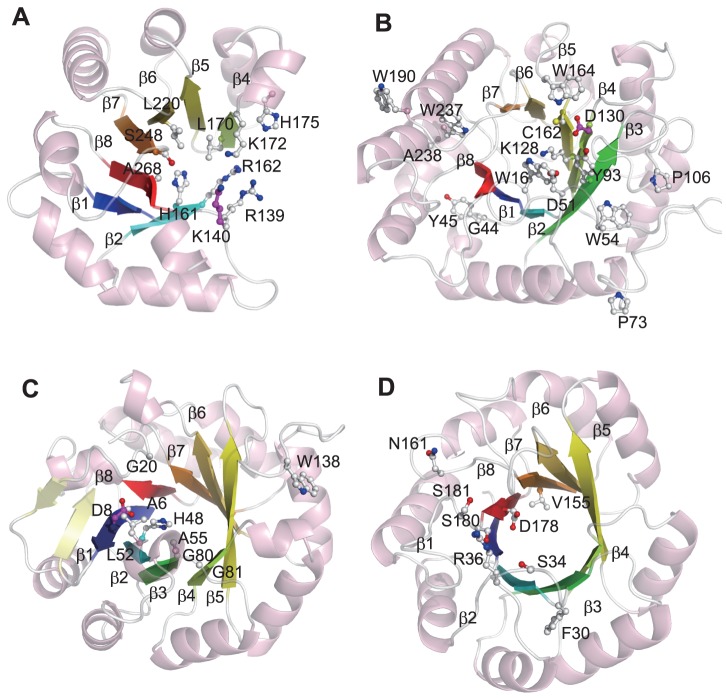
The rf-SDRs for (A) quinolinate phosphoribosyltransferase (hQPRTase; EC 2.4.2.19, CATH domain: 1qprF02), (B) α-galactosidase (α-Gal; EC 3.2.1.22, CATH domain: 1uasA01), (C) phosphoribosylformimino-5-aminoimidazole carboxamide ribonucleotide isomerase (HisA) (EC 5.3.1.16, CATH domain: 1qo2A00) and (D) phosphoribosylanthranilate isomerase (TrpF) (EC 5.3.1.24, CATH domain: 1nsjA00) in aldolase class I superfamily (CATH 3.20.20.70). The rf-SDRs are represented by balls and sticks, where nitrogen atoms are colored blue, oxygen atoms are red, sulfur atoms are yellow and carbon atoms are white. The carbon atoms of the active sites selected as rf-SDRs are colored magenta. Eight β-strands in a conventional barrel are colored blue, cyan, green, lemon, yellow, yelloworange, orange, and red, from the N-terminal to the C-terminal. The rf-SDRs in the figures A and B clearly show that the rf-SDRs for hQPRTase include the phosphate binding motif located in β-7 and β-8 in the conventional barrel structure but those for α-Gal are mainly located after β-1 to -5. The figure D shows the residues interacting with different moieties in substrates between HisA and TrpF, Ser 34 and Arg 36.

Figures 8A and 8B show the rf-SDRs of quinolinate phosphoribosyltransferase (hQPRTase; EC 2.4.2.19) and α-galactosidase (α-Gal; EC 3.2.1.22) as examples of enzymes having dissimilar functions. The rf-SDRs of hQPRTase included one core residue of the phosphate binding motif [60] Ala 268 at the end of β-10, which corresponds to β-8 in a conventional (α/β)_8_ barrel (in Figure 8A, the numbering of the β-strands based on the conventional barrel), and one of the catalytic residues, Lys 140 on β-1. Leu 170 and Lys 172 on β-4, the conformational change of which was suggested to be important for the specificity and reaction mechanism [61], were also included (Figure 8A). On the other hand, α-Gal recognizes the substrate having no phosphate moiety, mainly around the C-terminal ends of β-3 to β-6 [62]. In addition to the nucleophile Asp 130 at the end of β-4, many LBRs on these β-strands were selected as rf -SDRs (Figure 8B).

Figures 8C and 8D show ProFAR isomerase (HisA) (EC 5.3.1.16) and phosphoribosylanthranilate (PRA) isomerase (TrpF) (EC 5.3.1.24) as examples of enzymes having more similar functions. These enzymes catalyze the Amadori rearrangements of different substrates ProFAR and PRA by similar mechanisms [63], [64]. These substrates share a ribose-5-phosphate moiety, and ProFAR has an additional ribose connected by imidazole and PRA has an anthranilate moiety. Also known are PriA, which can catalyze both reactions, and its close homologue subHisA, which lacks the TrpF activity [65].

In the rf-SDRs of HisA, the only known catalytic residue (Asp 8) was selected. In TrpF, the corresponding active site, Cys 7, was not selected and the reason is unclear. In LBRs, some residues interacting with different moieties of each substrate were selected to be rf-SDRs: Ser 34 and Arg 36 of TrpF, which interact with the anthranilate moiety of the substrate [66], Gly 20 and Leu 52 of HisA, which would interact with the imidazole and attached amide moieties (inferred from the homologous PriA structure). Additionally, the rf-SDRs included His 48 and Trp 138 of HisA, likely to be important for the catalytic activity for PRA (also inferred from the PriA structure) [67]. In addition to these residues, different residues in different enzymes were selected, from those interacting with common parts of the substrates such as the phosphate moiety.

#### Phosphoenolpyruvate-binding domain superfamily (CATH 3.20.20.60)

The phosphoenolpyruvate-binding domain superfamily mainly consists of transferases (EC 2) and lyases (EC 4). Most of these enzymes have substrates or cofactors with a phosphate-moiety, while the phosphate binding sites are distributed over the C-terminal ends of β-strands 2 to 6. The predictors for six different enzymes consisting of two phosphotransferases with paired acceptors (EC 2.7.9), two oxo-acid-lyases (EC 4.1.3) and other transferases (EC 2) were constructed (Table S3). This superfamily was classified into the group of medium functional diversity.

Despite generally dissimilar active sites among these enzymes (Figure 6, light gray bars), the proportion of ASRs to be selected as rf-SDRs (23.5%) was lower than the average for the group of superfamilies with medium functional diversity (43.4%) (Tables S9 and S11). This result may be explained by the conservation of some of the active site residues. For example, pyruvate phosphate dikinase (EC 2.7.9.1) has the only known active site, Cys 831 [68] and this position in the alignment was also occupied by cysteine in pyruvate water dikinase (EC 2.7.9.2) (although no active site information is available for the latter enzyme). This position was not selected to be an rf-SDR, decreasing the average proportion of ASRs to be selected.

#### α/β-hydrolase superfamily (CATH 3.40.50.1820)

α/β-hydrolase superfamily is one of the large superfamilies, containing a wide variety of enzymes such as carboxylic acid ester hydrolases, peptidases, lipid hydrolases and haloalkane dehalogenases. In our dataset, predictors for 13 enzymes were constructed (Table S3). All these enzymes shared the first digit of the EC number (EC3; hydrolases) and this superfamily belonged to the group of superfamilies with medium functional diversity. A variety of functions are achieved by the conserved catalytic triad: a nucleophile (Ser, Cys or Asp) positioned after β-5, an acidic residue after β-7 and histidine after the last β-8 strand, and the versatile substrate binding sties by insertions and deletions at the C-terminal ends ofβ-3, 4, 6, 7 or 8 [69], [70]. Such a conserved catalytic triad and a similar chemical reaction mechanism are reflected in the proportion of ASRs to be selected as rf-SDRs (26.2%), which was lower than the average value (43.4%) for the group of medium functional diversity (Tables S9 and S11).

**Figure 9:**
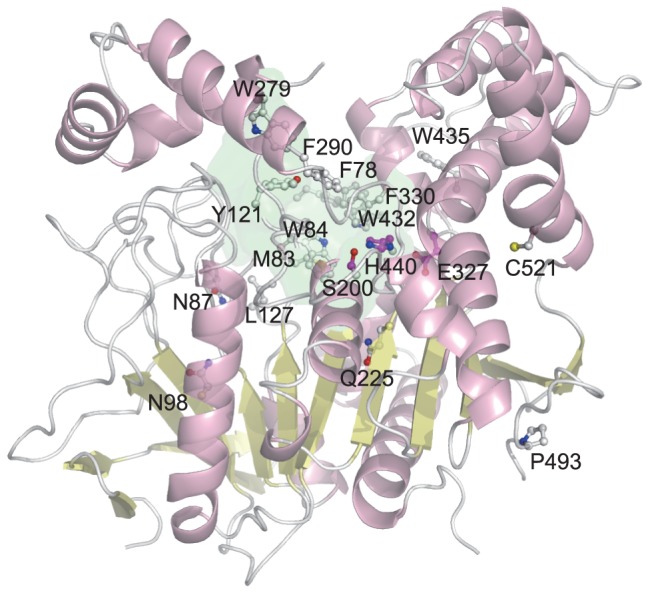
The rf-SDRs for acetylcholine esterase (AChE, EC 3.1.1.7, CATH domain: 1w76B00) in α/β-hydrolase superfamily (CATH 3.40.50.1820). The rf-SDRs are represented by balls and sticks, where carbon atoms are colored white, nitrogen atoms are blue, oxygen atoms are red and sulfur atoms are yellow. The active site gorge is partially represented by green surface. At the bottom of the active site gorge, the catalytic triads, which are not selected to be the rf-SDRs, are represented by balls and sticks and colored magenta. Many rf-SDRs are positioned around the catalytic gorge region.

For instance, acetylcholine esterase (AChE, EC 3.1.1.7) shown in Figure 9 has the conventional catalytic triad, Ser, Glu, and His, and a deep and narrow cavity around the catalytic site called “active site gorge” formed by large insertions, which is considered to determine the specificity for acetylcholine [71]. In 15 rf-SDRs, no residue of the catalytic triad was selected and about 40% of the rf-SDRs were located in the active site gorge. Trp 84 and Phe 330 are known as the anionic site to bind the choline moiety and Tyr 121, Trp 279 and Phe 290 are important for determining the gorge conformation [72]–[75]. Phe 290 causes steric hindrance with a large acyl group in the acyl pocket and plays a critical role in stabilizing the methyl moiety of acetylcholine [76].

These examples show whether each residue can be selected as an rf-SDR or not depends on whether it is conserved within a superfamily regardless of what roles the equivalent residues play in other enzymes. A residue may be conserved and used as a catalytic residue for the same chemical reaction in other enzymes and thus, it tends not to be selected as an rf-SDR, as observed in the glycosidase superfamily. A conserved residue may be used for catalyzing different chemical reaction but because of its conservation, it cannot be selected to be an rf-SDR, as observed in the α/β-hydrolase superfamily. In some superfamilies, different amino acid residues are used for catalyzing different chemical reactions or binding different ligands, in which case, these functional residues can be selected for rf-SDRs, as observed in the aldolase class I superfamily.

## Conclusion

We have developed EFPrf, a novel method based on random forests for predicting enzyme functions at the fourth-digit level of the EC number in each CATH homologous superfamily. As input attributes, we used amino acid residue similarities at ASRs, LBRs and CSRs, in addition to similarity in the full-length sequence. The prediction performance of EFPrf improved significantly over the decision trees constructed using BLAST scores alone (the simple model), especially in the low MTTSI regions, where it is known to be difficult to distinguish detailed functions by sequence similarity alone. This observation suggested that the information about functionally important sites would be useful for predicting detailed functions. During the construction of EFPrf, we also obtained the rf-SDRs from the most highly contributing attributes. The analysis of the selected superfamilies showed that the rf-SDRs included many experimentally verified SDRs. Moreover, we showed that the rf-SDRs reflected the mechanisms of functional diversification within each superfamily; the rf-SDRs both indicate a general degree of functional diversity (as measured by the proportion of ASRs to be selected as rf-SDRs) and the specific characteristics of each superfamily represented by the conservations of each residue in a superfamily. Thus, EFPrf is a useful tool for predicting detailed enzyme functions and the rf-SDRs are a good resource for determining SDRs by experimental and computational methods and understanding functional diversity in a superfamily.

In this paper, we examined individual domain sequences pre-assigned to a CATH superfamily for validating EFPrf. In practice, enzyme sequences often consist of multiple domains and in the future, we will develop a method for combining prediction results for the individual domains of a query sequence and producing an overall function prediction. In recent years, many methods have been proposed for predicting protein functions described by GO terms [13]. Our method can be extended to GO term prediction and may be efficient in the low sequence similarity region, where GO terms are also difficult to predict [24], [77].

## Materials and Methods

### Dataset preparation

Figure 2 shows an outline of the dataset construction. From the UniProtKB/Swiss-Prot database [39] (release 2010_06), we selected the enzyme sequences that: i) had been annotated with complete four-digit EC numbers, ii) were not fragment sequences and iii) had domains assigned to CATH [38] superfamilies in the Gene3D database [40]. A total of 332,021 enzyme domain sequences were obtained. In the following, an enzyme sequence refers to a protein domain sequence thus created, which was associated with a single CATH superfamily. The domain sequences were treated as independent sequences, although some of these were obtained from single multi-domain proteins. In order to obtain structural information, the 72,993 enzymes in the CATH database (ver. 3.3) were added to the 332,021 enzyme sequences. In each enzyme (as distinguished by the four-digit EC number) in each superfamily, all these sequences were clustered at a 95% sequence identity cutoff by using blastclust [78]. Also for each enzyme, a single representative structure was selected as the CATH S-level representative structure with the longest sequence length and the highest resolution. In the 95%-identity cluster that included the representative structure, the corresponding sequence was considered the representative of the cluster and in the other 95%-identity clusters, the longest sequence was selected as the representative. After the removal of redundancy, 201,708 sequences remained.

In the remaining sequences, a predictor was constructed for an enzyme if: 1) the enzyme belonged to a superfamily that contained at least one other enzyme in it, 2) the enzyme had a representative structure and ten or more sequences and 3) a total of ten or more sequences were available for the other enzymes as negative data in the superfamily. We randomly selected 80% of the sequences from a given enzyme and 80% of the sequences from the other enzymes in the superfamily for training. The remaining 20% of the sequences were used as a test dataset. A total of 1121 enzymes over 306 CATH homologous superfamilies were selected for benchmarking.

### Calculations of attributes for classifiers

In addition to the BLAST [14], [15] bit score, we used two types of scores as attributes: the scores calculated by using a full-length sequence and the scores at the functionally important positions in the alignment of a query sequence to a representative structure. The functionally important positions were defined to be the active sites, ligand binding sites and conserved site residues. In the following sections, we describe the selection of these positions and the score calculations.

#### Determination of the alignment positions used for attribute calculations

i) Active site and ligand binding residue positions from the literature and structural information: We obtained the literature information about active site residues from the Enzyme Catalytic-Mechanism Database (EzCatDB, ver. 20100722) [79] and the Catalytic Site Atlas (CSA, ver. 2.2.12) [45] database. All annotations in the EzCatDB and the original, hand-annotated entries derived from the primary literature in the CSA were used.

Ligand (substrate, cofactor, intermediate, products and their analogues) information in the Protein Data Bank (PDB) [80] was obtained from the EzCatDB and PROCOGNATE (ver. 1.6) [81] databases. All annotations in the EzCatDB and the cognate ligand entries with similarity scores higher than 0.5 in PROCOGNATE were used. Ligand binding residues were defined from complex structures by using LIGPLOT [82]. The residues that interacted with the ligands through both hydrogen bonds and hydrophobic interactions were considered as ligand binding residues. Ligand assignments to obsolete PDB entries were ignored.

We defined active site and ligand binding positions of each enzyme as the alignment positions, which were used by at least one PDB entry corresponding to that enzyme as an active site or a ligand-binding site, respectively. The position used as both active and ligand binding sites was defined to be an active site residue (ASR) position. The ASRs and ligand binding residues (LBRs) were mapped on to the representative structure for the calculation of attributes based on a multiple structural alignment, generated by MUSTANG [83], between the available complex structures and the representative.

ii) Conserved amino acid residue positions: For each enzyme in the training dataset, a multiple sequence alignment was generated by clustalw [84] and this alignment was aligned to the representative structure by FUGUE [41]. FUGUE performs sequence-structure comparison by utilizing environment-specific substitution tables (ESSTs). An ESST-based structural profile was calculated for the representative structure of each enzyme. To examine amino acid conservation, the entropy *S_k_* for each alignment position *k* was calculated as 
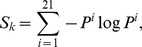
where *i* represents 20 types of amino acids plus a gap and *P^i^* is the fraction of amino acid type *i* at this position. The top 10% conserved residue positions (CBRs) in one enzyme were selected for the calculation of attributes. The positions where the fraction of the gap was above 20% were excluded from the entropy calculation. If the positions selected as CBRs were already defined as ASRs or LBRs, those positions were defined to be ASRs or LBRs.

Position-specific scoring matrices (PSSMs) [43] were also calculated from the multiple sequence alignments. The PSSM scores at the *i*th alignment positions were given by
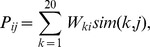
where *i* is the alignment position, *j* and *k* are the amino acid types and *sim*(*k*, *j*) is the score in the BLOSUM 62 matrix between amino acid types *j* and *k* [42]. The logarithmic weight W*_ki_* was defined, depending on occurrences of amino acid type *k* at position *i*, as
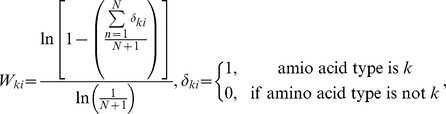
where *N* is the number of sequences in the alignment.

#### Calculation of scores

Given a query sequence, a BLAST search was performed against the sequences in the training dataset for each enzyme in each superfamily. The bit score for the top hit was used as an attribute for the predictors (see below). In the training mode, the bit score for the top hit, except for its own sequence, was used.

The other attributes were calculated based on an alignment between the query sequence and the representative structure by using three different scoring matrices: BLOSUM62, ESSTs and PSSMs. The latter two matrices were specific to each enzyme, as described in the previous section. The full-length sequence scores and the scores at ASRs, LBRs and CBRs were calculated.

### Construction of predictors and evaluation of performance

Decision trees were constructed by C4.5 [85] algorithms implemented in WEKA, a data mining software tool in Java (ver. 3.6.5) [86], with default parameters. Forests of decision trees were constructed by the random forests [31] algorithm implemented in R (ver. 2.15.1), a language and environment for statistical computing [87]. The default value was used for the number of attributes to split on at each node (floor(√ *n*), where *n* is the number of input attributes), since the number of attributes was different for each enzyme. The number of trees constructed for each classifier was set to be 500, by comparing averaged out-of-bag (OOB) error rates obtained from the models with 250, 500 and 750 trees (data not shown). In construction of random forest for each enzyme, the importance score for each attribute was calculated. We selected the top 3*floor(√*n*) ranked attributes as highly contributing attributes, analyzed their properties and defined the associated residues as random forest-derived specificity determining residues (rf-SDRs).

In order to evaluate prediction performance in regions where sequence identities between test and training sequences are low, we calculated the maximal test to training sequence identity (MTTSI) following Arakaki *et al.* [4] (see the reference for the detailed definition of MTTSI). Table S12 shows the number of positive and negative sequences in each MTTSI bin of the test set. Given a predictor for enzyme EC *a.a.a.a*, a set of prediction results were obtained (by using the test sequences) and these results were divided into eight bins according to their MTTSI values. Then for each bin, precision = TP/(TP+FP) and recall = TP/(TP+FN) were calculated, where TP is the number of true positives, FP is the number of false positives and FN is the number of false negatives. Finally, these precision and recall values were averaged over all the enzymes, for which it was possible to define the performance measure (i.e., (TP+FP) >0 for precision and (TP+FN) >0 for recall within a bin).

### Functional entropy of a superfamily

For classifying superfamilies at the EC third-digit level, we defined the functional entropy *S_func_* for each superfamily as follows:
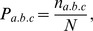


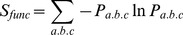
where *n_a.b.c._* is the number of predictors that share the first three digits of their EC numbers (*a.b.c*) and *N* is the total number of predictors in the superfamily. Using the functional entropy, superfamilies were classified into three groups: highly diverged (1.5≤*S_func_*), moderately diverged (0.5≤*S_func_*<1.5) and least diverged (0≦*S_func_*<0.5). The cutoff values were determined such that the occurrences of distinct EC numbers at the third-digit level within each superfamily approximately corresponded to one, two to four, and more than four, respectively (data not shown).

## Supporting Information

Figure S1 Distribution of the number of enzyme predictors constructed in a superfamily. The region between 20 to 70 is expanded and represented in the figure. Fifteen superfamilies contained more than ten enzyme predictors and the largest superfamily was NAD(P)-binding Rossmann-like domain superfamily (CATH 3.40.50.720) with 65 predictors.
(EPS)

Figure S2 Distribution of the active site residues (ASRs) and ligand binding residues (LBRs) in all superfamilies. The white bars represent the ASRs and the light gray bars represent the LBRs.
(EPS)

Figure S3 Distributions of fractions of the rf-SDRs in active site residues (ASRs, A) and ligand binding residues (LBRs, B), observed in the superfamilies with low, medium and high degrees of functional diversity classified at the fourth-digit level of EC numbers. The top and bottom of a box indicate 75th and 25th percentiles and the horizontal line in a box represents the median value. The top and bottom whiskers represent 90th and 10th percentiles.
(EPS)

Table S1 Number of predictors in each CATH homologous superfamily.
(XLSX)

Table S2 Precision and recall of enzymes in each MTTSI bin.
(DOCX)

Table S3 Prediction performance of each predictor.
(XLSX)

Table S4 List of the rf-SDRs.
(XLSX)

Table S5 Differences of scoring matrices selected in the rf-SDRs.
(DOCX)

Table S6 Classifications of superfamilies at the third- and forth-digit levels of EC numbers.
(XLSX)

Table S7 Averaged prediction performance for different classes of functional diversity at the third-digit level of EC numbers.
(DOCX)

Table S8 Averaged prediction performance for different classes of functional diversity at the forth-digit level of EC numbers.
(DOCX)

Table S9 The average proportion of ASRs/LBRs to be selected as rf-SDRs for different classes of functional diversity at the third-digit level of EC numbers.
(DOCX)

Table S10 The average proportion of ASRs/LBRs to be selected as rf-SDRs for different classes of functional diversity at the fourth-digit level of EC numbers.
(DOCX)

Table S11 The number of rf-SDRs in ASRs, LBRs and CSRs.
(DOCX)

Table S12 The number of positive and negative queries in each MTTSI bin.
(DOCX)
