# Supplementary material for: Prediction of Detailed Enzyme Functions and Identification of Specificity Determining Residues by Random Forests
Source: PLoS One. 2014 Jan 8;9(1):e84623. doi: 10.1371/journal.pone.0084623 (PMC3885575; doi:10.1371/journal.pone.0084623)
Supplement: Table S2 — Precision and recall of enzymes in each MTTSI bin. (DOCX) [file pone.0084623.s005.docx]

Table S2. Precision and recall of enzymes in each MTTSI bin

|  | Simple | | | | EFPrf | | | |
| --- | --- | --- | --- | --- | --- | --- | --- | --- |
| MTTSI | **Num^†^** | **Recall*** | **Num^†^** | **Precision*** | **Num^†^** | **Recall*** | **Num^†^** | **Precision*** |
| 0 (0-30%) | 122 | 0.38 (0.08) | 133 | 0.33 (0.08) | 122 | 0.30 (0.08) | 51 | 0.78 (0.11) |
| 1 (30-40%) | 272 | 0.66 (0.05) | 262 | 0.70 (0.05) | 272 | 0.67 (0.05) | 215 | 0.88 (0.04) |
| 2 (40-50%) | 469 | 0.82 (0.03) | 450 | 0.85 (0.03) | 469 | 0.81 (0.03) | 411 | 0.95 (0.02) |
| 3 (50-60%) | 605 | 0.92 (0.02) | 604 | 0.92 (0.02) | 605 | 0.90 (0.02) | 573 | 0.96 (0.01) |
| 4 (60-70%) | 660 | 0.95 (0.02) | 653 | 0.96 (0.01) | 660 | 0.92 (0.02) | 629 | 0.98 (0.01) |
| 5 (70-80%) | 772 | 0.97 (0.01) | 762 | 0.98 (0.01) | 772 | 0.95 (0.01) | 750 | 0.99 (0.01) |
| 6 (80-90%) | 847 | 0.97 (0.01) | 833 | 0.99 (0.00) | 847 | 0.96 (0.01) | 831 | 1.00 (0.00) |
| 7 (90-100%) | 779 | 0.98 (0.01) | 772 | 0.99 (0.01) | 779 | 0.98 (0.01) | 774 | 0.99 (0.01) |

* 95% confidence intervals in parentheses

† The number of enzymes, for which recall/precision was calculated.
