# Supplementary material for: Prediction of Detailed Enzyme Functions and Identification of Specificity Determining Residues by Random Forests
Source: PLoS One. 2014 Jan 8;9(1):e84623. doi: 10.1371/journal.pone.0084623 (PMC3885575; doi:10.1371/journal.pone.0084623)
Supplement: Table S5 — Differences of scoring matrices selected in the rf-SDRs. (DOCX) [file pone.0084623.s008.docx]

Table S5. Differences of scoring matrices selected in the rf-SDRs

| Superfamily | BLOSUM62 | ESSTs | PSSMs |
| --- | --- | --- | --- |
| rf-SDRs | 6851 | 6436 | 7596 |
| non rf-SDRs | 16758 | 17532 | 16345 |
