# Supplementary material for: Prediction of Detailed Enzyme Functions and Identification of Specificity Determining Residues by Random Forests
Source: PLoS One. 2014 Jan 8;9(1):e84623. doi: 10.1371/journal.pone.0084623 (PMC3885575; doi:10.1371/journal.pone.0084623)
Supplement: Table S8 — Averaged prediction performance for different classes of functional diversity at the forth-digit level of EC numbers. (DOCX) [file pone.0084623.s011.docx]

Table S8. Averaged prediction performance for different classes of functional diversity at the forth-digit level of EC numbers

| Class^*^ | Precision | Recall | F-measure |
| --- | --- | --- | --- |
| Low | 0.97 | 0.90 | 0.94 |
| Medium | 0.97 | 0.91 | 0.94 |
| High | 0.97 | 0.84 | 0.90 |
