# Supplementary material for: Prediction of Detailed Enzyme Functions and Identification of Specificity Determining Residues by Random Forests
Source: PLoS One. 2014 Jan 8;9(1):e84623. doi: 10.1371/journal.pone.0084623 (PMC3885575; doi:10.1371/journal.pone.0084623)
Supplement: Table S9 — The average proportion of ASRs/LBRs to be selected as rf-SDRs for different classes of functional diversity at the third-digit level of EC numbers. (DOCX) [file pone.0084623.s012.docx]

Table S9. The average proportion of ASRs/LBRs to be selected as rf-SDRs for different classes of functional diversity at the third-digit level of EC numbers

| Class | Proportion of ASRs/% | Proportion of LBRs/% |
| --- | --- | --- |
| Low | 35.0 | 41.4 |
| Medium | 43.4 | 42.6 |
| High | 43.7 | 40.2 |
