# Supplementary material for: Prediction of Detailed Enzyme Functions and Identification of Specificity Determining Residues by Random Forests
Source: PLoS One. 2014 Jan 8;9(1):e84623. doi: 10.1371/journal.pone.0084623 (PMC3885575; doi:10.1371/journal.pone.0084623)
Supplement: Table S11 — The number of rf-SDRs in ASRs, LBRs and CSRs. (DOCX) [file pone.0084623.s014.docx]

Table S11. The number of rf-SDRs in ASRs, LBRs and CSRs

| Superfamily | ASR | LBR | CSR |
| --- | --- | --- | --- |
| 3.20.20.80 | 34/104 (32.7%) | 95/279 (34.1%) | 142/416 (34.17%) |
| 3.20.20.60 | 4/17 (23.5%) | 19/49 (38.8%) | 40/127 (31.5%) |
| 3.20.20.70 | 70/135 (51.9%) | 133/436 (30.5%) | 193/608 (31.7%) |
| 3.40.50.1820 | 16/61 (26.2%) | 50/149 (33.6%) | 98/264 (37.1%) |

Numerators mean the number of the ASRs/LBRs in a superfamily and denominators mean the number of the ASRs/LBRs to be selected as the rf-SDRs.
