# Supplementary material for: Prediction of Detailed Enzyme Functions and Identification of Specificity Determining Residues by Random Forests
Source: PLoS One. 2014 Jan 8;9(1):e84623. doi: 10.1371/journal.pone.0084623 (PMC3885575; doi:10.1371/journal.pone.0084623)
Supplement: Table S12 — The number of positive and negative queries in each MTTSI bin. (DOCX) [file pone.0084623.s015.docx]

Table S12. The number of positive and negative queries in each MTTSI bin

| MTTSI | Positive | Negative |
| --- | --- | --- |
| 0 (0-30%) | 183 | 28215 |
| 1 (30-40%) | 565 | 246 |
| 2 (40-50%) | 1310 | 241 |
| 3 (50-60%) | 2446 | 115 |
| 4 (60-70%) | 3538 | 67 |
| 5 (70-80%) | 4724 | 72 |
| 6 (80-90%) | 6581 | 45 |
| 7 (90-100%) | 4456 | 34 |
